# Soluble programmed death-ligand 1 (sPDL1) and neutrophil-to-lymphocyte ratio (NLR) predicts survival in advanced biliary tract cancer patients treated with palliative chemotherapy

**DOI:** 10.18632/oncotarget.12810

**Published:** 2016-10-21

**Authors:** Hyerim Ha, Ah-Rong Nam, Ju-Hee Bang, Ji-Eun Park, Tae-Yong Kim, Kyung-Hun Lee, Sae-Won Han, Seock-Ah Im, Tae-You Kim, Yung-Jue Bang, Do-Youn Oh

**Affiliations:** ^1^ Department of Internal Medicine, Seoul National University Hospital, Seoul, Korea; ^2^ Cancer Research Institute, Seoul National University College of Medicine, Seoul, Korea

**Keywords:** soluble PDL1, PDL1, immunotherapy, biliary tract cancer, biomarker

## Abstract

Programmed death-ligand 1 (PD-L1) expression in tumor tissue is under investigation as a candidate biomarker in immuno-oncology dug development. The soluble form of PD-L1 (sPDL1) is suggested to have immunosuppressive activity. In this study, we measured the serum level of sPDL1 and evaluated its prognostic implication in biliary tract cancer (BTC). Blood was collected from 158 advanced BTC patients (68 intrahepatic cholangiocarcinoma, 56 gallbladder cancer, 22 extrahepatic cholangiocarcinoma and 12 ampulla of vater cancer) before initiation of palliative chemotherapy. Serum sPDL1 was measured using an enzyme-linked immunosorbent assay. Clinical data included neutrophil-to-lymphocyte ratio (NLR), platelet-to-lymphocyte ratio (PLR) and systemic immune-inflammation index (SII, neutrophil × platelet/lymphocyte). The patients were assigned to two cohorts (training and validation cohort) using a simple random sampling method to validate the cut-off value of each marker. Validation was performed using a twofold cross-validation method. Overall survival (OS) of all patients was 9.07 months (95% CI: 8.20-11.33). Median sPDL1 was 1.20 ng/mL (range 0.03-7.28, mean 1.50, SD 1.22). Median NLR, PLR and SII were 2.60, 142.85 and 584.93, respectively. Patients with high sPDL1 (≥0.94 ng/mL) showed worse OS than patients with low sPDL1 (7.93 vs. 14.10 months, HR 1.891 (1.35-2.65), *p*<0.001). In multivariate analysis, high sPDL1 and NLR were independent poor prognostic factors. In conclusion, serum sPDL1 can be measured and has significant role on the prognosis of advanced BTC patients treated with palliative chemotherapy.

## INTRODUCTION

Biliary tract cancer (BTC) consists of intrahepatic cholangiocarcinoma (IHCC), extrahepatic cholangiocarcinoma (EHCC), gallbladder cancer (GB ca) and ampulla of vater cancer (AoV ca), and it has a poor prognosis. [1]

Gallstones, chronic hepatitis B or C, inflammatory disease of bile duct such as primary sclerosing cholangitis and certain liver parasites are well known risk factors of BTC, which might make the chronic inflammation state. [2-4]

The human immune system could recognize cancer cells and suppress cancer progression or metastasis. However, cancer cells have innate or acquired immune evasion mechanisms, which result in disease progression. [5, 6] Immune-related cells including neutrophils, lymphocytes and platelets could suppress the circulating tumor cells. [7, 8] Recently, immuno-oncology has become a promising approach in the field of new anti-cancer drug development, especially with the success of immune checkpoint inhibitors in melanoma, non-small cell lung cancer and renal cell carcinoma. [9-12]

In BTC, there is also a strong potential to use immune modulation strategy to fight against cancer. [13-15] In the genomic spectra of BTC, unsupervised clustering of global gene expression levels determined by transcriptome sequencing revealed 4 molecular subgroups. [13] Among these, the worst prognostic group was enriched with genes involved in the immune system and cytokine activity. Hypermutated cases were significantly enriched in this subgroup, and the expression of immunosuppressive immune checkpoint molecules including programmed death-ligand 1 (PD-L1) (CD274) was significantly higher in this worst prognosis subgroup. In total, 45.2% of cases showed an increase in the expression of immune checkpoint molecules. This subgroup may be a good target population for immunotherapy.

For these immunotherapies, many candidate biomarkers such as Wnt inhibitor Dickkopf-1, IFN-γ and TGF-β are under investigation. [14, 15] PD-L1 expression in cancer cells or tumor microenvironment is a candidate biomarker for immune checkpoint inhibitors. [16-19] PD-L1 is expressed in macrophages and dendritic cells and can bind to programmed death-l (PD1) in activated T and B lymphocytes and NK cells. [20] When PD-L1 binds to PD1, inhibitory signals are activated, which in turn act as a negative regulator of T cell activity in tissue. [21, 22] Tumor cells also express PD-L1 to activate inhibitory signals as a mechanism of overcoming host immune response, so-called immune checkpoints. [23, 24]

For assessment of PD-L1 expression on tumor cells or immune cells, acquisition of tumor tissue in invasive measure is needed, which is always a challenging issue, especially in BTC. The soluble form of PD-L1 (sPDL1) in peripheral blood is believed to impair host immunity and cause poor clinical outcomes in renal cell carcinoma, diffuse large B cell lymphoma and multiple myeloma. [25-27]

The purpose of this study was to measure sPDL1 in the serum of BTC patients and to evaluate its clinical implication in advanced BTC patients receiving palliative chemotherapy.

## RESULTS

### Patient characteristics

In the entire cohort (N = 158), median age was 59.6 years old (range, 31.3- 76.2) and primary sites of tumor were: IHCC 43.0%, GB ca 35.4%, EHCC 13.9% and AoV ca 7.6% (Table 1).

Median follow-up duration was 95.3 months, and median OS was 9.07 months (95% CI: 8.20- 11.33) (Supplementary Figure 1).

A total of 143 patients received 5-FU based chemotherapy. Among them, 140 patients received combined chemotherapy with 5-FU and platinum. Three patients were treated with 5-FU+doxorubicin+ mitomycin C. Fifteen patients received gemcitabine with platinum combination chemotherapy.

Using a simple random sampling method, 79 patients were randomly assigned to the training cohort and the other 79 patients to validation cohort to validate biomarkers. Median OS was 8.23 months (95% CI: 6.80-11.30) and 10.13 months (95% CI: 8.53-13.60) in the training cohort and validation cohort, respectively (Supplementary Figure 1). The clinical characteristics such as age, sex, primary origin, albumin, CEA, CA19-9, etc. did not show significant differences between cohorts.

**Table 1:** Patient characteristics

	Training cohort	Validation cohort	Training cohort vs validation cohort	Entire cohort
	N = 79 (%)	N = 79 (%)	*P*-value	N=158 (%)
Follow up	117.5(65.8-NA)	94.2(91.0-NA)	0.163	95.3 (91.0-NA)
Age				
Median(range)	60.0(37.2-76.2)	59.6(31.3-73.6)		59.6 (31.3- 76.2)
Sex			0.867	
Male	47 (59.5)	56 (70.9)		103 (65.2)
Female	32 (40.5)	23 (29.1)		55 (34.8)
Diagnosis			0.357	
IHCC	35 (44.3)	33 (41.8)		68 (43.0)
EHCC	10 (12.7)	12 (15.2)		22 (13.9)
AoV ca	4 (5.1)	8 (10.1)		12 (7.6)
GB ca	30 (38.0)	26 (32.9)		56 (35.4)
Disease extent			0.404	
Initially unresectable	55 (69.6)	48 (60.6)		103 (65.2)
Recurrent	24 (30.4)	31 (39.2)		55 (34.8)
Total bilirubin			0.299	
Normal	58 (77.3)	55 (76.4)		113 (76.9)
Elevated	17 (22.7)	17 (23.6)		34 (23.1)
Albumin			0.520	
Decreased	10 (12.7)	13 (16.7)		23 (14.6)
Normal	69 (87.3)	65 (83.3)		134 (85.4)
CEA			0.911	
Normal	48 (60.8)	56 (71.8)		104 (65.4)
Elevated	31 (39.2)	22 (28.2)		53 (33.8)
CA-19-9			0.376	
Normal	28 (35.4)	31 (39.7)		59 (37.6)
Elevated	51 (64.6)	47 (60.3)		98 (62.4)
Survival				
Alive	3 (3.8)	3 (3.8)	1.000	6 (3.8)
Death	76 (96.2)	76 (96.2)		152 (96.2)

IHCC, intrahepatic cholangiocarcinoma; EHCC, extrahepatic cholangiocarcinoma; AoV ca, ampulla of vater cancer; GB ca, gallbladder cancer; CEA, carcinoembryonic antigen; CA-19-9, carbohydrate antigen 19-9.

Normal value; total bilirubin, 0.2- 1.2 mg/dL; albumin, 3.3-5.2 g/dL; CEA 0-5 ng/dL; CA-19-9, 0-37 U/mL

### Biomarkers for host immunity

Median sPDL1 of enrolled patients was 1.20 ng/mL (range, 0.5-2.1) (Table 2). Median sPDL1 of normal healthy populations was 1.20 ng/mL (range, 0.5- 1.9) which was similar to BTC patients’ serum level (Supplementary Figure 2). There are no significant difference of sPDL1 according to age and gender between healthy population and BTC cancer patients (Supplementary Table 1A and 1B).

**Figure 1:**
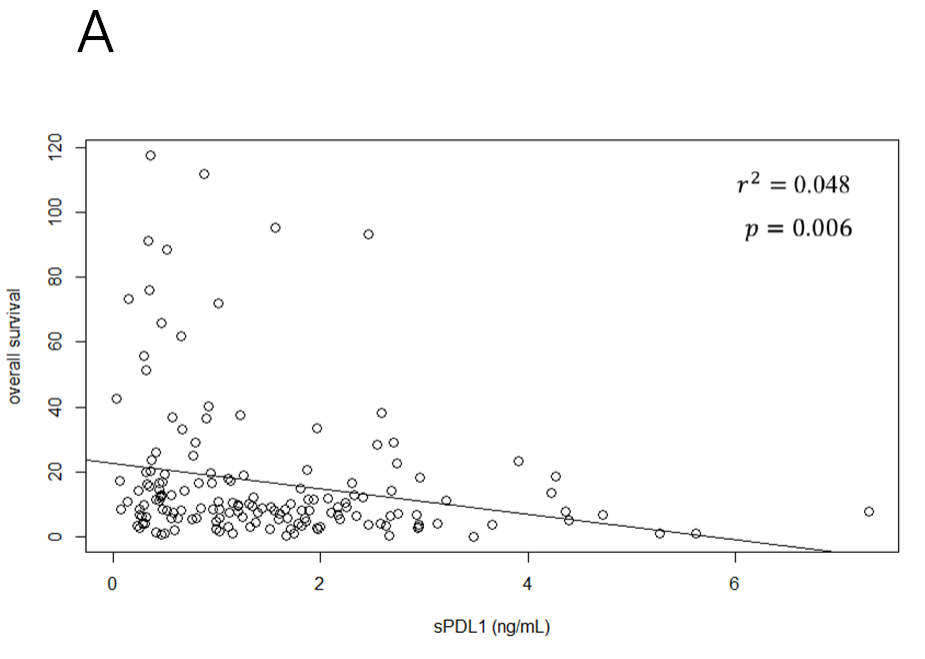
sPDL1 and overall survival **A.** Correlation between sPDL1 and overall survival: Each dot represents a patient. Patients with high levels of sPDL1 were distributed in the shorter overall survival group. **B.** Cubic splines model between sPDL1 and overall survival: sPDL1 shows an increased hazard ration with increasing level. Hazard ratio is represented by a solid line and 95% confidential interval is a dotted line.

Median sPDL1 according to disease extent differed in patients: 1.44 ng/mL (range, 0.03-7.28) in initially unresectable cases and 0.94 ng/mL (range, 0.06-4.39) in recurrent cases (*p* = 0.005). According to primary origin, level of sPDL1 was highest in IHCC and lowest in EHCC. The patients with lower sPDL1 showed more prolonged OS (Figure 1A). Analysis of cubic splines between hazard ratio and sPDL1 showed an increased risk of death with increasing level of sPDL1 (Figure 1B).

**Table 2:** Biomarkers for host immunity

	Training cohort	Validation cohort	Training cohort vs validation cohort	Entire cohort
	N = 79 (%)	N = 79 (%)	*P*	N=158 (%)
**sPDL1**				
Median(range) (ng/mL)	1.11(0.06-7.28)	1.35(0.03-4.39)	0.356	1.20 (0.03-7.28)
Cut-off	2.94	1.02		0.94
< cut-off	70(88.6)	30(38.0)		61(38.6)
≥cut-off	9(11.4)	49(62.0)		97(61.4)
Disease extent; median (range)				*P*=0.005
Initially unresectable	1.31(0.08-7.28)	1.73(0.03-4.26)	0.344	1.44(0.03-7.28)
Recurrent	0.62(0.06-2.94)	1.01(0.24-4.39)	0.420	0.94(0.06-4.39)
Diagnosis; median (range)				*P*=0.015
IHCC	1.44(0.08-5.62)	1.74(0.24-4.26)	0.436	1.56(0.08-5.62)
EHCC	0.64(0.30-1.23)	0.82(0.25-4.39)	0.767	0.72(0.25-4.39)
AoV ca	0.78(0.06-2.41)	1.41(0.03-3.47)	0.683	1.23(0.03-3.47)
GB ca	1.13(0.15-7.28)	1.27(0.30-3.91)	0.737	1.20(0.15-7.28)
**NLR**			0.440	
Median(range)	2.58(0.71-17.60)	2.63(0.64-10.22)		2.60 (0.64-17.60)
Cut-off	3.80	3.45		3.45
< cut-off	53(67.1)	53(68.0)		105(66.5)
≥cut-off	26(32.9)	25(32.1)		52(32.9)
**PLR**			0.997	
Median(range)	142.85(50.27-476.66)	144.49(6.60-449.74)		142.85 (6.60-476.66)
Cut-off	89.62	91.82		89.62
< cut-off	13(16.5)	8(10.3)		21(13.3)
≥cut-off	66(83.5)	70(90.0)		136(86.1)
**SII**			0.408	
Median(range)	631.10(106.25-6195.20)	577.42(44.75-2926.13)		584.93 (44.75-6195.20)
Cut-off	826.89	499.63		572.38
< cut-off	52(65.8)	30(38.5)		75(47.5)
≥cut-off	27(34.2)	48(61.5)		82(51.9)

NLR, neutrophil-to-lymphocyte ratio; PLR, platelet-to-lymphocyte ratio; SII, systemic immune-inflammation index; sPDL1, soluble form PD-L1.

**Figure 2:**
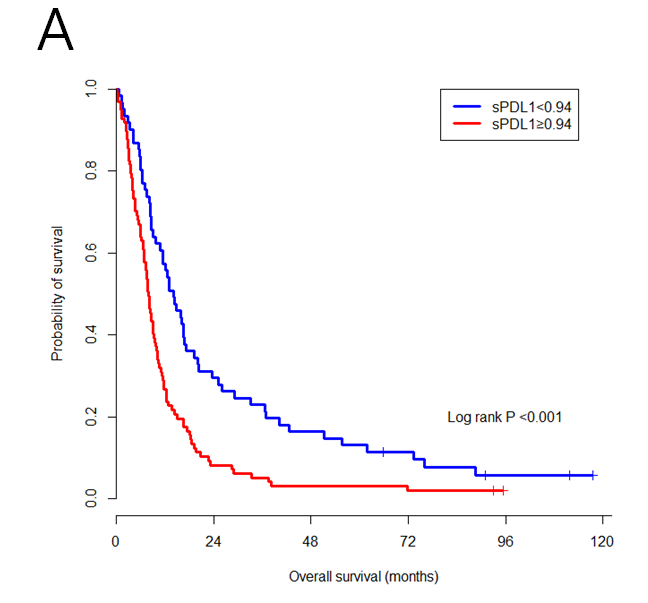
Survival outcomes according to sPDL1 **A. and NLR B.** The patients with a high level of sPDL1 or NLR had worse overall survival.

The cut-off value of sPDL1 in entire cohort to separate different prognosis groups, calculated by the minimum P value approach, was 0.94 ng/mL. The sPDL1 cut-off value in training and validation cohorts was 2.94 and 1.02 ng/mL, respectively, and it was analyzed for validation of sPDL cut-off in entire cohort. The *p* value of sPDL1 cross-validation was 0.024, meaning that the patients with sPDL1 ≥0.94 ng/mL had worse OS than sPDL1 <0.94 ng/mL (Table 3, Figure 2A).

Median NLR was 2.60 (range, 0.64-17.60), PLR was 142.85 (range, 6.60-17.60) and SII was 584.93 (44.75-6195.20). Cut-off values of NLR, PLR and SII to separate OS were 3.45, 89.62 and 572.38, respectively (Table 2), and NLR was the only biomarker with significant *p* value in cross-validation, as shown in Table 3. The patients with lower NLR than the cut-off showed longer OS (*p* < 0.001) (Figure 2B). Cubic splines model between hazard ratio and each marker of NLR, PLR and SII also showed a positive relation (Supplementary Figure 3A-3C).

**Table 3:** Validation of cut-off value

	Training		Validation		Cross validation *P* value
	Cut-off	HR (95% CI)	Cut-off	HR (95% CI)	
sPDL	2.94	3.988(1.73-9.17)	1.02	1.777(1.04-3.03)	0.024
NLR	3.80	2.721(1.49-4.97)	3.45	2.920(1.58-5.40)	0.009
PLR	89.62	1.939(0.99-3.81)	91.82	1.711(0.79-3.70)	0.091
SII	826.89	2.458(1.35-4.47)	499.63	1.859(1.09-3.19)	0.193

sPDL, soluble form of PD-L1; OS, overall survival; HR, hazard ratio; CI, confidence interval.

### Prognostic factors for OS

In univariate analysis, performance status, diagnosis, disease extent (initially unresectable *vs*. recurrent), number of metastasized organs, low albumin and high CEA/CA-19-9 were significant prognostic factors (Table 4). Among immune-related biomarkers, sPDL1, NLR and SII were significant for OS (HR (95% CI): sPDL1, 1.891 (1.35-2.65), *p* < 0.001; NLR, 2.604 (1.84-3.69), *p* < 0.001; and SII, 1.917 (1.38-2.66), *p* < 0.001). sPDL1 and NLR were also significant independent prognostic factors in multivariate analysis along with disease extent, albumin and CEA.

**Table 4:** Prognostic factors for OS

	Univariate analysis		Multivariate analysis	
	HR(95% CI)	*P*	HR(95% CI)	*P*
Age ≥ 60 years (vs. <60)	1.324(0.96-1.82)	0.957		
ECOG PS ≥ 2 (vs. 0-1)	2.565(1.04-6.32)	**0.040**	0.743(0.29-1.92)	0.539
Relapsed disease (vs. unresectable)	0.502(0.35-0.71)	**<0.001**	0.601(0.41-0.88)	**0.009**
No. of metastasis organ >2 (vs. ≤2)	1.574(1.08-2.30)	**0.018**	0.855(0.56-1.31)	0.474
Total bilirubin > 1.2 (vs. ≤1.2)	0.759(0.51-1.13)	0.167		
Albumin > 3.3 (vs. ≤3.3)	0.399(0.25-0.63)	**<0.001**	0.560(0.34-0.92)	**0.023**
CEA > 5 (vs. ≤5)	1.860(1.32-2.63)	**<0.001**	1.684(1.16-2.44)	**0.006**
CA-19-9 > 37 (vs. ≤37)	1.585(1.13-2.22)	**0.007**	1.362(0.96-1.94)	0.086
sPDL1 > 0.94 (vs. ≤0.94)	1.891(1.35-2.65)	**<0.001**	1.565(1.07-2.30)	**0.023**
NLR > 3.45 (vs. ≤3.45)	2.604(1.84-3.69)	**<0.001**	2.048(1.26-3.34)	**0.004**
PLR > 89.62 (vs. ≤89.62)	1.581(0.99-2.52)	0.052		
SII > 572.38 (vs. ≤572.38)	1.917(1.38-2.66)	**<0.001**	0.928(0.59-1.45)	0.745

ECOG PS, Estern Cooperative Oncology Group Performance Status; UNL, upper normal limit; LNL, lower normal limit; CEA, carcinoembryonic antigen; CA-19-9, carbohydrate antigen 19-9; NLR, neutrophil-to-lymphocyte ratio; PLR, platelet-to-lymphocyte ratio; SII, systemic immune-inflammation index; sPDL1, soluble form of PD-L1; OS, overall survival; HR, hazard ratio; CI, confidence interval.

Normal value; total bilirubin, 0.2- 1.2 mg/dL; albumin, 3.3-5.2 g/dL; CEA 0-5 ng/dL; CA-19-9, 0-37 U/mL

## DISCUSSION

This study demonstrated that sPDL1 could be measured by ELISA in the serum of BTC patients and that high level of sPDL1 was a negative prognostic factor in BTC patients who received palliative chemotherapy. NLR and SII which represent a host immune-inflammation state were also significant prognostic factors for survival in univariate analysis, and only NLR was significant in multivariate analysis.

PD1/PD-L1 is associated with an inflammatory tumor microenvironment as a regulator of inhibitory signals, and its expression could be a candidate biomarker for patient selection for anti-PD1/PD-L1 monoclonal antibodies. [28, 29] To evaluate PD1/PD-L1 in tumor cells and tumor microenvironment, the acquisition of adequate tissue is obligatory. Tumor biopsy is a challenge in the management of cancer patients. Especially in BTC, some types of tumor spread only along the bile duct, which make it difficult to get adequate tumor tissue. To overcome such issue, the liquid biopsy or liquid biomarker is an appealing approach to pursue.

PD-L1 is expressed on the surface of activated antigen presenting cells. It is also expressed on the membrane of human tumor cells, which is one of the mechanisms of escaping the host immune system. sPDL1 is thought to be a circulating biologically active protein which is released from PD-L1-positive tumor cells or immune cells. It could contribute to systemic immunosuppression. [25, 26]

However, the exact function and role of sPDL1 is mainly unresolved so far. In the current study, we measured sPDL1 by ELISA in the serum of BTC patients, which was collected before initiation of palliative chemotherapy. Importantly, sPDL1 could be measured in all patients, whose range was 0.03-7.28 ng/mL. The recurrent cases, which are believed to have low tumor burden, showed a lower level of sPDL1 than initially unresectable cases. In parallel with this sPDL1 level, OS was longer in recurrent cases than initially unresectable cases (14.20 month *vs* 7.93 months). Among the 4 origins of BTC, IHCC showed the highest level of sPDL1 and worst OS (8.32 months).

The level of sPDL1 is an independent poor prognostic factor for OS in univariate and multivariate analysis. The patients with sPDL1 <0.94 ng/mL had significantly prolonged OS which was validated by the twofold cross-validation method in training and validation cohorts. The level of sPDL1 before chemotherapy could be a useful biomarker to predict prognosis of BTC patients.

Recently, several studies on the antitumor action of the host immune have shown that NLR, PLR and SII have prognostic value in solid tumors including BTC. [30-32] We also analyzed the association between OS and immune biomarkers such as NLR, PLR and SII. According to the cut-off in each cohort, patients with a high level of NLR, PLR and SII had worse OS. When we validated the cut-off value of these immune markers, NLR was the only validated one with adverse prognostic value. On multivariate analysis, a high level of NLR implied prognostic impact on OS.

Our study had limitations. It was a retrospective, single center study. The patients’ serum collected for biomedical research before initiation of palliative chemotherapy was retrospectively analyzed. Even though we tried to validate the cut-off value of biomarkers, these findings should be further validated in a different independent patient cohort with prospective design. Furthermore, studies on the function of sPDL1 and its mechanism of action are needed. In parallel, as a prognostic factor, the more strength of sPDL1 compared with NLR, which is a very pragmatic, easily applicable clinical factor for the practice on advanced biliary tract cancer patients, should be studied. Besides the study on prognostic role of sPDL1, the study on the predictive role of sPDL1 to the chemotherapy efficacy is also interesting topic and needed to reveal the biology of sPDL1.

Despite the limitations, a prognostic impact of the host immune-inflammation system in BTC patients was uncovered in our study. To the best of our knowledge, this is the first study on the role of sPDL1 in BTC patients. In addition, analysis was performed in a relatively large cohort and by dividing the respective cohort for more exact validation.

In conclusion, sPDL1 can be measured in the serum of BTC patients, and a high level of sPDL is an independent prognostic factor in advanced BTC patients treated with palliative chemotherapy. This study suggests the possible role of sPDL1 as a liquid biomarker for immuno-oncology drug development.

## PATIENTS AND METHODS

### Patients and data collection

In a patient cohort with pathologically diagnosed, unresectable or recurrent BTC, patients whose blood samples were collected before initiation of palliative first-line chemotherapy were considered for this study. Between 2004 and 2009, patients who provided informed consent for the biomarker analysis study in Seoul National University Hospital were finally recruited for this study (N = 158).

Data of baseline demographics were collected from medical charts. Laboratory data including total bilirubin, albumin, CEA, CA-19-9 and blood cell count with neutrophils, platelets and lymphocytes were also collected. Neutrophil-to-lymphocyte ratio (NLR) and platelet-to-lymphocyte ratio (PLR) were calculated by dividing the neutrophil or platelet count by the lymphocyte count obtained before initiation of first-line palliative chemotherapy. Systemic immune-inflammation index (SII) was defined as neutrophil x platelet/lymphocyte count. [31]

For evaluating the sPDL1 levels in normal healthy population, we did the age-, and sex-matched selection of normal control to the biliary tract cancer patients in a ratio of 3:1. We used the serum samples obtained from healthy population, which had been collected under IRB-approved studies (IRB number: 1103-150-357).

sPDL1 was measured using an enzyme-linked immunosorbent assay (PDCD1LG1 ELISA kit, USCN Life Science) in patient and normal healthy person's serum according to the manufacturer's instructions. Each sample was analyzed in duplicate. [25]

### Statistical analysis

Pearson's chi-square test or Fisher's exact test was used for categorical variables, as appropriate. A *t*-test was used for comparison of means. Overall survival (OS) was defined as the time from day 1 of chemotherapy to date of death or last follow-up. Median OS was calculated using the Kaplan-Meier method and comparisons of difference between groups was assessed using log-rank tests. We defined patients’ current status on death or alive using National Mortality Tracking System at the data cut-off timing. There was no one censored case for overall survival.

Univariate and multivariate analysis for OS was performed using Cox regression models. Factors with *p* < 0.05 in univariate analysis were collected and analyzed in multivariate regression models. All statistical tests were two-sided, with significance defined as *p* < 0.05.

We randomly divided enrolled patients into two cohorts (training and validation cohort) using a simple random sampling method. [33] Cut-off values of each variable (sPDL1, NLR, PLR and SII) for OS prediction were determined by using the minimum *p* value approach in each group. [34] To validate the cut-off value of each biomarker, a twofold cross-validation method was used. [35] The training and validation cohort were divided into a higher and lower group according to cut-off value. If there were significant differences on survival analysis between the high and lower group, the cut-off value of the entire cohort was defined significant prognostic value.

We performed this analysis using SAS 9.2 (by SAS Institute Inc., Cary, NC, USA)

### Ethics

The study protocol was reviewed and approved by the Institutional Review Board of Seoul National University Hospital (H-1408-039-600). All studies were conducted according to guidelines for biomedical research (Declaration of Helsinki).

## SUPPLEMENTARY MATERIALS TABLES AND FIGURES


