# Supplementary material for: Soluble programmed death-ligand 1 (sPDL1) and neutrophil-to-lymphocyte ratio (NLR) predicts survival in advanced biliary tract cancer patients treated with palliative chemotherapy
Source: Oncotarget. 2016 Oct 21;7(47):76604–12. doi: 10.18632/oncotarget.12810 (PMC5363533; doi:10.18632/oncotarget.12810)
Supplement: Supplementary file 1 [file oncotarget-07-76604-s001.pdf]

Soluble programmed death-ligand 1 (sPDL1) and neutrophil-to-lymphocyte ratio (NLR) predicts survival in advanced biliary tract cancer patients treated with palliative chemotherapy

Supplementary Material

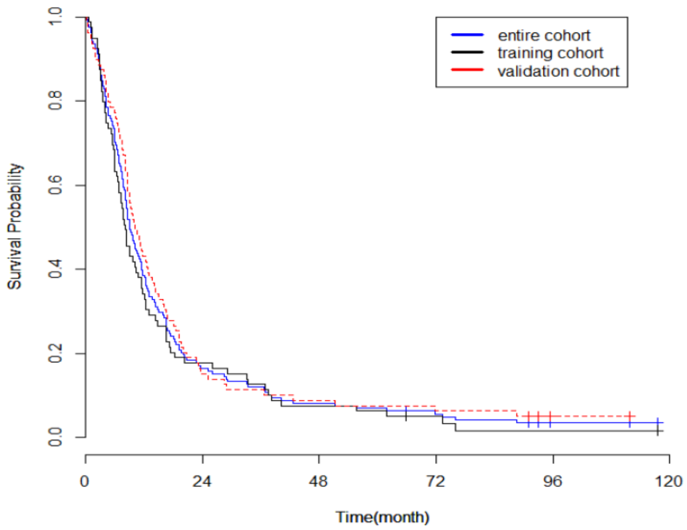

Sup.Fig1 Kaplan-Meier curve for overall survival

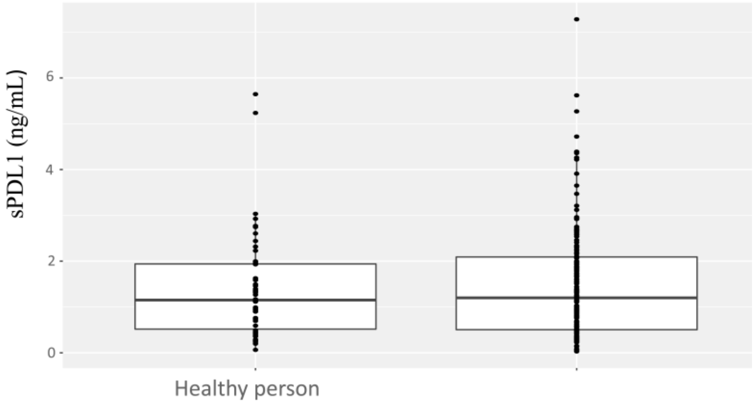

Sup.Fig2 Comparison sPDL1 between healthy person and cancer patients

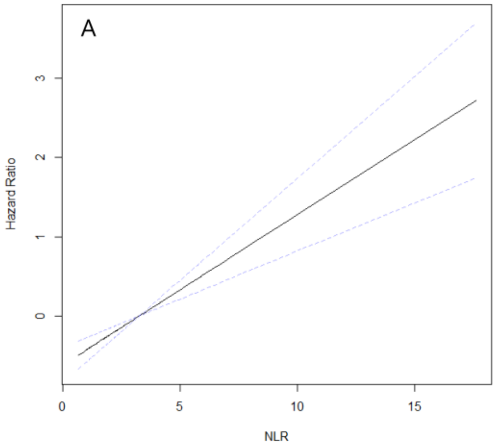

Sup.Fig3A Cubic splines models between hazard ration for death and NLR

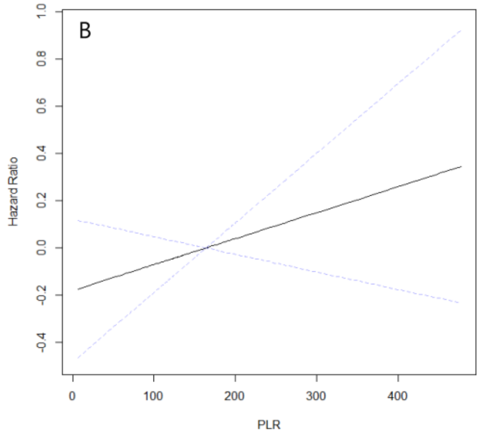

Sup.Fig3B Cubic splines models between hazard ration for death and PLR

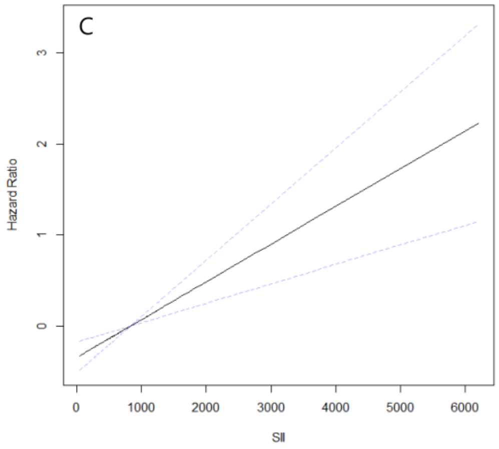

Sup.Fig3C Cubic splines models between hazard ration for death and SII

Supplementary Table 1. sPDL1 according to age(A) and gender(B)

(A) sPDL1 according to age

age	40's			50's			60's			70's		
Group	Healthy (N=8)	Cancer pt (N=25)	*P*	Healthy (N=18)	Cancer pt (N=58)	*P*	Healthy (N=20)	Cancer pt (N=61)	*P*	Healthy (N=4)	Cancer pt (N=14)	*P*
sPDL1	0.9 [0.3; 1.7]	1.4 [0.6; 2.4]	0.223	1.6 [1.1; 2.3]	1.2 [0.5; 2.1]	0.153	1.0 [0.5; 1.4]	1.1 [0.5; 2.2]	0.443	0.6 [0.3;0.8]	1.0 [0.5;1.8]	0.190

(B) sPDL1 according to gender

gender	Male				Female			
Group	Healthy (N=32)	Cancer (N=103)	Total (N=135)	*P*	Healthy (N=18)	Cancer (N=55)	Total (N=73)	*P*
sPDL1	1.1 [0.5; 1.6]	1.3 [0.6; 2.4]	1.2 [0.5; 2.2]	0.255	1.4 [0.7; 2.3]	1.2 [0.5; 1.7]	1.2 [0.5; 1.8]	0.474

`
